# Developing Early Markers of Cognitive Decline and Dementia Derived From Survey Response Behaviors: Protocol for Analyses of Preexisting Large-scale Longitudinal Data

**DOI:** 10.2196/44627

**Published:** 2023-02-21

**Authors:** Haomiao Jin, Doerte U Junghaenel, Bart Orriens, Pey-Jiuan Lee, Stefan Schneider

**Affiliations:** 1 School of Health Sciences University of Surrey Guildford United Kingdom; 2 Center for Economic and Social Research University of Southern California Los Angeles, CA United States; 3 Center for Self-Report Sciences University of Southern California Los Angeles, CA United States; 4 Department of Psychology University of Southern California Los Angeles, CA United States

**Keywords:** dementia, mild cognitive impairment, early markers, survey response behaviors, epidemiology

## Abstract

**Background:**

Accumulating evidence shows that subtle alterations in daily functioning are among the earliest and strongest signals that predict cognitive decline and dementia. A survey is a small slice of everyday functioning; nevertheless, completing a survey is a complex and cognitively demanding task that requires attention, working memory, executive functioning, and short- and long-term memory. Examining older people’s survey response behaviors, which focus on how respondents complete surveys irrespective of the content being sought by the questions, may represent a valuable but often neglected resource that can be leveraged to develop behavior-based early markers of cognitive decline and dementia that are cost-effective, unobtrusive, and scalable for use in large population samples.

**Objective:**

This paper describes the protocol of a multiyear research project funded by the US National Institute on Aging to develop early markers of cognitive decline and dementia derived from survey response behaviors at older ages.

**Methods:**

Two types of indices summarizing different aspects of older adults’ survey response behaviors are created. Indices of subtle reporting mistakes are derived from questionnaire answer patterns in a number of population-based longitudinal aging studies. In parallel, para-data indices are generated from computer use behaviors recorded on the backend server of a large web-based panel study known as the Understanding America Study (UAS). In-depth examinations of the properties of the created questionnaire answer pattern and para-data indices will be conducted for the purpose of evaluating their concurrent validity, sensitivity to change, and predictive validity. We will synthesize the indices using individual participant data meta-analysis and conduct feature selection to identify the optimal combination of indices for predicting cognitive decline and dementia.

**Results:**

As of October 2022, we have identified 15 longitudinal ageing studies as eligible data sources for creating questionnaire answer pattern indices and obtained para-data from 15 UAS surveys that were fielded from mid-2014 to 2015. A total of 20 questionnaire answer pattern indices and 20 para-data indices have also been identified. We have conducted a preliminary investigation to test the utility of the questionnaire answer patterns and para-data indices for the prediction of cognitive decline and dementia. These early results are based on only a subset of indices but are suggestive of the findings that we anticipate will emerge from the planned analyses of multiple behavioral indices derived from many diverse studies.

**Conclusions:**

Survey response behaviors are a relatively inexpensive data source, but they are seldom used directly for epidemiological research on cognitive impairment at older ages. This study is anticipated to develop an innovative yet unconventional approach that may complement existing approaches aimed at the early detection of cognitive decline and dementia.

**International Registered Report Identifier (IRRID):**

DERR1-10.2196/44627

## Introduction

Dementia, a syndrome characterized by a progressive cognitive decline that interferes with independent functioning, affects a large and growing number of older adults. With increases in life expectancy in the population, the individual, family, and societal burdens associated with dementia are expected to accelerate rapidly, making the disease one of the major public health challenges of this century [1]. It is increasingly recognized that the identification of preclinical markers that are predictive of the future transition from healthy cognition to dementia is of paramount importance [2,3]. Such markers could allow earlier intervention and even modest delays in disease onset or progression, which would have a substantial impact on incidence rates and health care costs.

Despite existing efforts to discover biomarkers of cognitive decline and dementia [4,5], there is a need to develop and investigate nonbiological, behavioral markers that can be turned to when using biomarkers is impractical [6]. Accumulating research has shown that subtle alterations in daily functioning, for example, telephone or computer use, managing finances, and completing forms, are among the earliest and strongest signals that predict cognitive decline and dementia [4,5,7-9]. Reductions in the efficiency, speed, and consistency of performing these and other cognitively demanding activities can be observed up to a decade before diagnosis [3,7]. Accordingly, recommendations by the US National Institute on Aging and Alzheimer’s Association highlight the need to identify and validate new neurobehavioral measures, including assessments of very early deficits in functional performance, that could detect preclinical manifestations of the disease [2,3].

We propose to develop indicators of functioning that can be gleaned from the way older people complete survey assessments, that is, questionnaires and computer-administered assessments. Surveys are ubiquitous in research and clinical practice, and many large-scale national and international longitudinal panel surveys on aging are presently in the field. A survey is a small slice of everyday functioning; nevertheless, completing a survey is a complex and cognitively demanding task that requires attention, working memory, executive functioning, and short- and long-term memory. Research on the psychology of survey methods confirms that answering survey questions requires considerable cognitive effort: respondents are expected to interpret the meaning of each question, retrieve relevant information from memory, integrate it into a summary judgment, and map their judgment onto the provided response alternatives [10]. As a result, examining older people’s survey response behaviors, which focus on how respondents complete surveys irrespective of the content being sought by the questions, may represent a valuable but often neglected resource that can be leveraged to develop behavior-based early markers of cognitive decline and dementia that are cost-effective, unobtrusive, and scalable for use in large population samples.

The purpose of this paper is to describe the study protocol of the “Testing Early Markers of Cognitive Decline and Dementia Derived from Survey Response Behaviors” study. The study has the following 3 specific aims:

1. Aim 1 will develop questionnaire answer patterns and para-data indices of potential functional deficits based on survey response behaviors observed in a number of longitudinal aging studies.
2. Aim 2 will evaluate the validity and clinical utility of the indices and synthesize validity results across multiple longitudinal studies using meta-analytic principles.
3. Aim 3 will determine the optimal combination of survey response behavior indices for the prediction of subsequent mild cognitive impairment and dementia onset.

## Methods

### Overall Design

This study will develop early markers of cognitive decline and dementia from 2 types of indices summarizing different aspects of older adults’ survey response behaviors. One category is comprised of indices that can be directly computed from the pattern of answers in questionnaires. These questionnaire answer pattern indices can be generated from participants’ responses in a number of population-based longitudinal aging surveys, providing a repository of archival data that allows for predictions of current cognitive function from response behavior data collected many years ago. Examples of these questionnaire answer pattern indices are skipping questions, agreeing or disagreeing with statements regardless of content, errors due to nondifferentiation among questions or response options, or giving contradictory responses. The second category is comprised of so-called para-data indices, that is, auxiliary information unobtrusively collected alongside people’s responses in surveys that are collected electronically (eg, on the web). Examples of such para-data indices are participant response times for survey questions, mouse movements, and keystrokes.

### Ethics Approval

The study is approved by the University of Southern California Institutional Review Board (UP-22-00147) and the University of Surrey Research Integrity and Governance Office (FHMS 21-22 216 EGA).

### Data Sources

#### Data for Creating Questionnaire Answer Pattern Indices

Existing longitudinal studies on aging are screened to identify data sets suitable for creating questionnaire answer pattern indices. Inclusion criteria are (1) a minimum of 3 waves of data collection (to estimate change over time) in which (2) participants complete a minimum of 40 multi-item rating scale questions at each assessment occasion (to achieve a sufficient body of questionnaire answers to construct the indices), (3) respondents are 50 years of age or older (studies that include younger participants are truncated or only later waves are used), and (4) availability of objective tests of cognitive functioning. The criteria for inclusion in these studies are intentionally broad to enable the construction and examination of questionnaire answer pattern indices under a wide range of conditions. Excluded are studies that are cross-sectional, focus on younger adults, or administer questionnaires on a single occasion.

Table 1 shows the 15 longitudinal studies that we have identified as of October 2022 as eligible data sources for creating questionnaire answer pattern indices. The combined sample size in these studies is >50,000, including over 300,000 assessment time points. Multiple self-report scales of varying content are administered at each measurement occasion in all studies, including personality, mental health, attitudes, beliefs and values, quality of life, and emotional experience, which are used for generating the questionnaire answer pattern indices. Each study provides multiple cognitive functioning measures at each assessment wave, including the Mini-Mental State Examination [11], the Telephone Interview for Cognitive Status [12], and formal clinical assessments of dementia status.

**Table 1:** Eligible longitudinal aging studies for creating questionnaire answer pattern indices.

Study	First wave^a^	Sampling interval (years)	Waves, n	Minimum age (years)	Approximate sample size, n	Survey items (note^b^), n	Dementia status ascertained
Health and Retirement Study	2006	2	>6	50	12,000	>100	T^c^
English Longitudinal Study of Ageing	2002	2	>8	50	8000	>100	M^d^, T
Longitudinal Aging Study Amsterdam	1992	3	>7	55	3100	>100	M
Irish Longitudinal Study on Ageing	2010	2	>4	50	5000	>100	M, T
Memory and Aging Project	2004	1	>15	65	1700	60	C^e^, M
Australian Longitudinal Study of Ageing	1992	3-4	>7	65	1100	80	M
Canberra Longitudinal Study	1990	4	4	70	1000	50	C, M
Sydney Memory and Aging Study	2005	2	>7	70	1000	80	C, M
Minority Aging Research Study	2005	1	>14	65	950	60	C, M
Einstein Aging Study	2004	1	>7	70	800	80	C, M
Swedish Adoption or Twin Study of Aging	1986	3	>7	50	600	>100	C, M
Octogenarian Twins Study	1991	2	5	79	300	80	C, M
Longbeach Longitudinal Study	1994	3	5	50	150	40	C
Mexican Health and Aging Study	2012	3	>3	50	15,000	40	T
Survey of Health, Aging, and Retirement in Europe	2004	2	>7	50	20,000	40	M, T

^a^Years indicate the first assessment wave to be used in the analyses.

^b^Minimum number of available individual questions from multi-item rating scales at each assessment occasion.

^c^T indicates that dementia status is ascertained by validated cutoffs from Telephone Interview for Cognitive Status [13,14].

^d^M indicates that dementia status is ascertained by Mini-Mental State Exam cutoffs [15,16].

^e^C indicates that dementia status is ascertained by formal clinical assessment.

#### Data for Creating Para-Data Indices

To create para-data indices, para-data are identified from the backend server of the Understanding America Study (UAS). The UAS is a probability-based web-based panel launched in 2014 that comprises 10,000 panel members, of whom about 5000 are aged 50 years or older. Panel members are randomly selected through address-based sampling and are compensated based on the estimated time spent on each survey. Panel members without prior access to the internet are provided with a tablet computer and internet access. Since its launch, the UAS has conducted over 400 surveys of varying length. Details about the UAS can be found on the UAS website [17].

**Figure 1:**
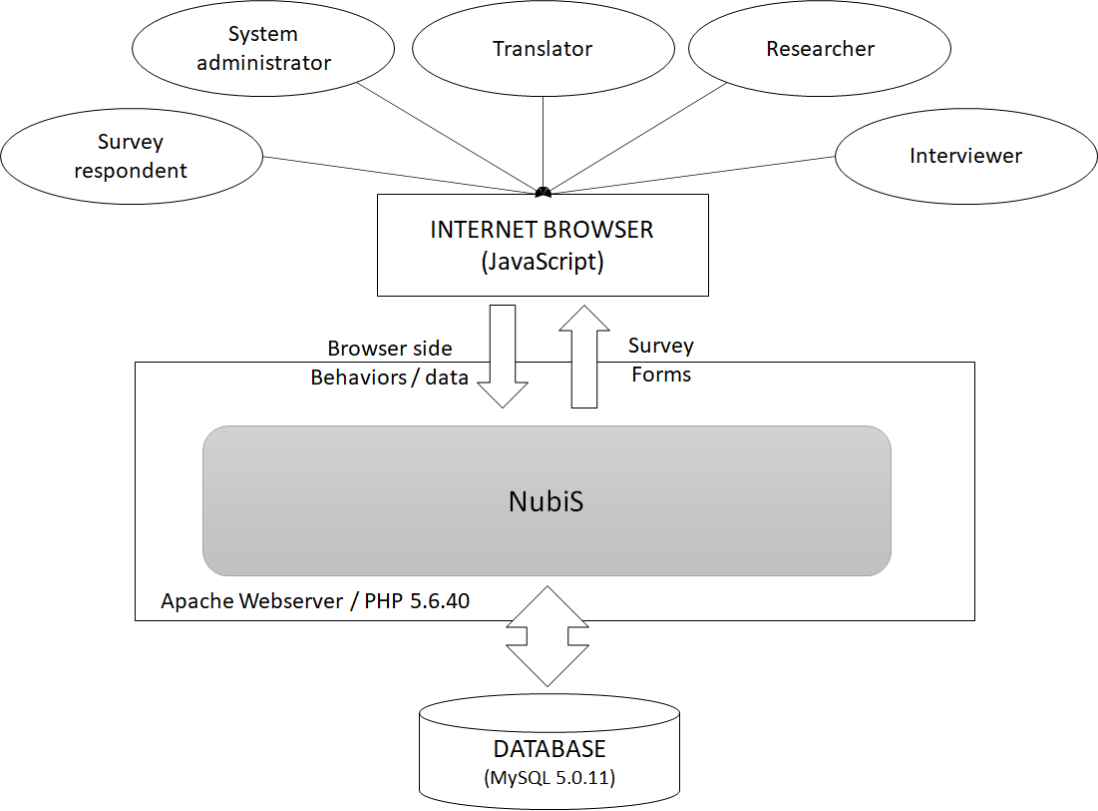
The system architecture of NubiS.

Surveys in the UAS are conducted and managed by using an internally developed web-based software called NubiS (Centre for Economic and Social Research, University of Southern California), whose development was co-led by author BO. Figure 1 illustrates the system architecture of NubiS. The software runs on an Apache Webserver 2.4.10 with PHP version 5.6.40 and provides a role-based web interface for user groups such as survey respondents and system administrators. NubiS has the capability to capture and store both survey responses and browser-side behaviors, including mouse clicks, keys pressed, errors, leave, return, or progress in surveys, and user platform information using JavaScript.

There are seven types of raw para-data pertaining to the survey response process on NubiS: (1) “timing” records the time spent per screen, defined as the time from which the server outputs the survey screen to the browser to the moment that the respondent uses the back or next button; (2) “error” records errors detected on the browser side; (3) “key pressed” records keys pressed on the browser side; (4) “mouse click” records mouse clicks or touch events on the browser side; (5) “leave/return to survey” records the leaving or returning to the browser tab that contains the survey; (6) “survey invitation/response” records survey nonresponse and the times of survey invitation, uptake, and completion; and (7) “platform information” records the type of device, operating system, and browser type of the device used by the respondent.

### Procedures for Creating Survey Response Behavior Indices

We periodically conduct literature searches in the areas of aging and cognition, psychometrics and survey methodology, computer use behaviors among older adults, and the psychology of survey response to identify publications describing questionnaire answer pattern indices and para-data indices that may be useful for research on cognitive decline and dementia. SAS macros are developed to compute the identified indices. For indices that require different analysis software, like R (R Foundation for Statistical Computing) or M*plus* (Muthén & Muthén), macros are developed to execute these external analysis steps in batch mode from within the SAS environment. The generated indices are matched to the survey waves or scales of each eligible aging study and stored for analysis. As of October 2022, we have identified a total of 20 questionnaire answer pattern indices (Table 2) and 20 paradata indices (Table 3). We will periodically refresh our literature search and update the list of response behavior indices.

**Table 2:** List of questionnaire answer pattern indices.

Index				Description
**Item nonresponse**				
		Proportion of skipped items [18]		This index measures the proportion of skipped items in a multi-item survey, which has been suggested to be associated with cognitive functioning [18].
**Response styles**				
		Acquiescence (“yea-saying”) Disaquiescence (“nay-saying”) Extreme response tendency Midpoint responses		These indices are based on detecting abnormal response styles to identify respondents with cognitive impairment. Emerging evidence has suggested that certain response styles, such as acquiescence and an extreme response tendency, may be predictive of cognitive impairment or associated with important risk factors for cognitive impairment like low education in older adults [19,20].
**Expressions of uncertainty**				
	Proportion of “don’t know” answers Response uncertainty parameter [21]		These indices capture the extent to which participants express uncertainty about their answers in the surveys.	
**Classical Test Theory indices of improbable responses**				
	Multivariate outlier rate [22] Person-total correlation [23] Psychometric synonyms [24] Psychometric antonyms [24]		These indices are based on detecting improbable or implausible responses to identify respondents with cognitive impairment. Cognitive impairment has been suggested to result in improbable answers [24].	
**Item Response Theory person-fit statistics**				
	Polytomous *l*_z_ index [25] Normed Guttman errors [26] *U*3 index [27]		These indices are also designed to detect improbable or implausible response patterns, based on modern test theory approaches.	
**Random errors or noise in responses**				
	Individual test reliability Inter-item standard deviation [28]		These indices capture unreliable answer patterns to capture random variability in attention or fluctuating cognitive performance.	
**Response inertia**				
	Residual autocorrelation across consecutive responses [29] Long-string analysis [24]		These indices measure carry-over effects in people’s response patterns, which may express an inability to adapt responses to changing question contents.	
**Response pattern drift within a survey**				
	Improvement or worsening in missing, stylistic, outlying, misfitting, or random response patterns within a survey Change-point analysis of weighted residuals [30]		These indices measure trends in response patterns over the course of a survey. Worsening response behaviors as a participant completes multiple questions in succession may indicate mental fatigue or lower sustained attention ability.	

**Table 3:** List of para-data indices.

Index			Description
**Survey completion**			
	Time lag from invitation to survey uptake Time of day survey was taken Missed survey Time from uptake to complete the survey	This category of indices measures aspects of survey completion, such as whether a survey is incomplete and the time spent on completing a survey. Emerging evidence suggests that these indices may be predictive of cognitive impairment [31].	
**Response time**			
	Median browser-side response time Deviations from the median browser-side response time Variability in response times across questions Autocorrelation (inertia) of response times across questions Item response theory person-fit statistics response time patterns Pause rate (no response for >20 seconds) Median pause duration	This category of indices measures aspects of response times such as the median response times, variability, and autocorrelations. A growing number of studies suggest that response times and their derived measures may be associated with cognitive impairment [32,33].	
**Errors and corrective behaviors**			
	Proportion of corrected or changed answers Rate of error messages received (eg, invalid entry) Rate of “back” button use Rate of “next” button use without a completed response	This category of indices measures aspects of errors and corrective behaviors in answering web-based surveys.	
**Mouse and touch efficiency**			
	Number of mouse clicks per page (median) [34] Variability of mouse clicks across screens [34] Total pixel count (inefficiency in movement: “wandering around on screen”) [34]	This category of indices measures aspects of mouse clicks and inefficiencies in mouse movement behaviors.	
**Keystrokes**			
	Median time between keystrokes (for text entries) [34] Variability of time between keystrokes (within a text entry) [34]	This category of indices measures temporal rhythms of keystrokes from keyboard entries by respondents for survey questions.	

### Data Analysis Procedures

#### Validity and Clinical Utility of the Proposed Indices

We will conduct in-depth examinations of the properties of the generated questionnaire answer pattern and para-data indices for the purpose of evaluating their concurrent validity, sensitivity to change, and predictive validity in each study, as well as the replicability and robustness of their performance across different studies and survey contents. We first evaluate the cross-sectional correlations between the survey response behavior indices and cognitive functioning scores. We then evaluate each generated index for its sensitivity to change in 3 aspects, including the average rate of change with age (ie, whether an index reflects a decline in performance with age), differential change with age (ie, the extent to which some people change more rapidly than others with age), and correlated changes (ie, the extent to which changes in an index correlate with changes in a cognitive test score). In addition, we evaluate prospective relationships between the generated indices and subsequent changes in cognitive functioning measures. We examine the lagged effects between the indices and subsequent cognitive test scores across the measurement occasions in each study. We also test the ability of the survey response behavior indices at baseline (ie, the first measurement occasion) to predict the later onset of dementia in time-to-event (survival) analyses. To ensure robustness and replicability of the variables’ performances, we repeat the analyses across all eligible longitudinal aging studies. As for the paradata indices, we repeat the analyses for different surveys in the UAS.

#### Synthesis of Indices Across Studies

Methods of individual participant data meta-analysis are used to analyze and aggregate results across different studies. These methods have been considered state-of-the-art for study comparison and integration, and the research team has successfully implemented them in prior research projects [35,36]. Given that the different panel studies vary in data structure (eg, number and spacing of measurement occasions) and do not administer exactly the same instruments (eg, cognitive functioning tests), we do not harmonize the data across studies or combine them into a single large data set. Instead, analyses are first carried out separately for each data set (ie, for each study) using identical data-analytic models. The resulting effect sizes are then synthesized across data sets using meta-analytic techniques. This method preserves the heterogeneity of effect sizes across studies, and, because it synthesizes effect sizes without directly pooling data into a single data set, it can accommodate the integration of results from diverse studies. Thus, individual participant data meta-analysis enables us to generate a cumulative evidence-base of the performance and validity of the survey response behavior variables with great efficiency.

#### Selection of the Optimal Combination of Indices

We use feature selection methods to determine the optimal subset of survey response behavior indices that are most predictive of subsequent dementia onset when combined with each other. Feature selection is a machine learning technique that is particularly well-suited for determining those indices that jointly optimize a prediction equation for individuals while identifying and discarding those indices that are irrelevant or provide redundant predictive information. Among the multiple approaches considered, we use a regularized survival modeling method that uses a path-based algorithm for the Cox proportional hazards model, regularized by elastic net penalties, to rank and select the most predictive indices [37]. The model penalizes the regression coefficients of the survival model, effectively causing many coefficient estimates to shrink to zero. The predictive power of each index is then ranked based on shrinkage speed, with the slowest shrinking index having the strongest predictive power. Overall model accuracy is assessed with common measures of model performance such as sensitivity, specificity, and area under the receiver-operating curve.

## Results

As mentioned in the previous section, we will have identified 15 longitudinal ageing studies as eligible data sources for creating questionnaire answer pattern indices by October 2022 (Table 1). We have identified a total of 20 questionnaire answer pattern indices (Table 2) and 20 paradata indices (Table 3). We have conducted a preliminary investigation to test the utility of the questionnaire answer pattern indices for the prediction of cognitive decline and dementia. The tested indices included the proportion of skipped items [18], the multivariate outlier rate [22], the normed Guttman errors [26], the inter-item standard deviation [28], and the person-total correlation [23] derived from the 2006/2008 waves in the Health and Retirement Study (N=12,554). Survival analyses (using the Fine and Gray competing-risk model [38]) were used for each index to examine whether it predicted dementia onset in the subsequent 8 years, while statistically controlling for age and education and while taking death into account as a competing event. Dementia status was ascertained using the criteria by Langa and Weir, which categorize respondents as cognitively normal, cognitively impaired (not dementia), or having dementia. The classification is based on cognitive test scores with cutoffs calibrated against in-person clinical assessments in the Aging, Demographics, and Memory Study [13]. It is not a clinical diagnosis but has proven useful for research purposes. As shown in Table 4, the hazard rates of dementia onset are between 1.52 and 2.25 times as high in respondents with questionnaire answer patterns that suggested cognitive problems (highest tertile) as for those in the lowest tertile (*P*<.001 for each index), and the middle tertile fell between the lowest and the highest. Details on the results of these analyses have been published elsewhere [39].

**Figure 2:**
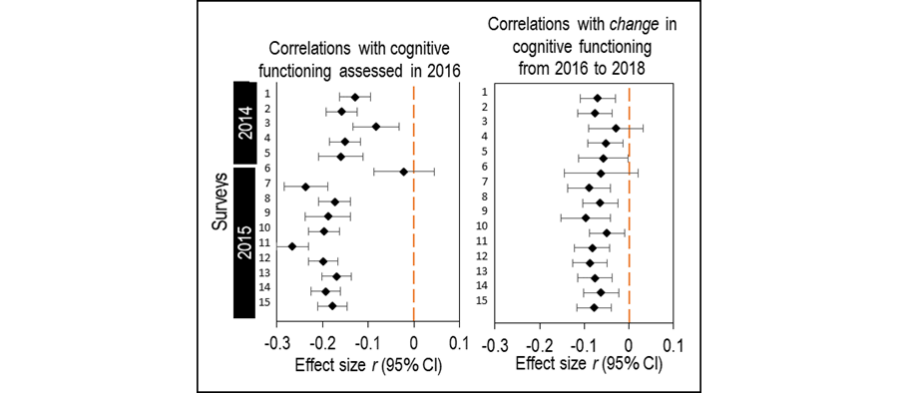
Relationships between survey response times and subsequent cognitive functioning in the Understanding America Study.

To assess the predictive power of the para-data indices, we derived participants’ response times to survey items in 15 UAS surveys that were fielded from mid-2014 to 2015. We examined whether they were predictive of subsequent cognitive functioning measured in 2016 and of cognitive function change from 2016 to 2018. The response times to UAS survey items were log-transformed to reduce skewness. Cognitive functioning was evaluated by using the verbal analogies test, an executive functioning and analogical reasoning task with good specificity and sensitivity in discriminating normal controls from probable dementia patients. The analysis included 3829 UAS panelists 50 years and older and examined both zero-order associations and associations controlling for age and education. As shown in Figure 2, longer response times on 14 of the 15 surveys were significantly correlated at the 0.05 level with lower subsequent cognitive test scores in 2016 as well as with worsening cognitive functioning from 2016 to 2018. The significance and direction of these associations did not change after controlling for age and education.

Notably, these early results are based on only a subset of questionnaire response patterns and para-data indices but are suggestive of the findings that we anticipate will emerge from the planned analyses of multiple behavioral indices derived from many diverse studies.

**Table 4:** Hazard ratios (95% CI) for associations between selected questionnaire answer pattern indices and subsequent dementia risk in the Health and Retirement Study (N=12,554).

Tertile for each variable	Proportion of skipped items	Multivariate outlier rate	Normed Guttman errors	Interitem SD	Person-total correlation
Low	Reference	Reference	Reference	Reference	Reference
Middle	1.34 (1.15-1.56)	1.38 (1.12-1.71)	1.43 (1.18-1.76)	1.28 (1.05-1.57)	1.40 (1.14-1.71)
High	1.52 (1.24-1.86)	2.25 (1.84-2.75)	2.09 (1.73-2.53)	1.93 (1.61-2.34)	2.03 (1.68-2.46)

## Discussion

This paper describes the protocol of a multiyear research project that aims to develop early markers of cognitive decline and dementia from survey response behaviors. The project plans to develop 2 types of indices, known as questionnaire answer pattern indices and paradata indices, summarizing different aspects of older adults’ survey response behaviors. The project will examine the psychometric properties of the created indices and create predictive models of cognitive decline and dementia using the indices. Preliminary results are suggestive of the predictive power of survey response behavior indices for forecasting cognitive decline and dementia. To the best of our knowledge, this study is the first multiyear funded project aspiring to develop nonbiological early markers of cognitive decline and dementia from large-scale survey response behavior data at older ages.

If successful, the study may open novel opportunities for advancing research on health care for cognitive decline and dementia. Indices derived from survey response behaviors that could serve as preclinical markers of cognitive decline could greatly enhance the usefulness of questionnaires collected in large-scale surveys for epidemiological research on the risk and protective factors of dementia. Questionnaires are also routinely collected in medical care settings, and it is possible that survey response behavior indices extracted in these settings could potentially supplement information from standardized cognitive tests. In clinical trial research, the Food and Drug Administration recommendations for early-stage Alzheimer disease trials encourage the development of novel approaches for the evaluation of preclinical functional deficits [40], and response behavior indices extracted from questionnaires administered in clinical trials might potentially supplement core clinical measures that serve as trial endpoints. Furthermore, the survey response behavior indices may be useful as a proxy for cognitive impairment in aging survey research where standardized cognitive functioning measures are not assessed.

Survey response behaviors are a relatively inexpensive data source, but they are seldom used directly for epidemiological research on cognitive impairment at older ages. This innovative yet unconventional approach may complement existing approaches aimed at the early detection of cognitive decline and dementia.

## Appendix

Multimedia Appendix 1 Peer-review report for the project.

## Abbreviations

UAS: Understanding America Study

## Data Availability

The Health and Retirement Study data supporting the findings in this paper are openly available in the study’s website [41]. The Understanding America Study data supporting the findings in this paper are openly available in the study’s website [17].
