# Supplementary material for: Developing Early Markers of Cognitive Decline and Dementia Derived From Survey Response Behaviors: Protocol for Analyses of Preexisting Large-scale Longitudinal Data
Source: JMIR Res Protoc. 2023 Feb 21;12:e44627. doi: 10.2196/44627 (PMC9993229; doi:10.2196/44627)
Supplement: Multimedia Appendix 1 [file resprot_v12i1e44627_app1.pdf]

SUMMARY STATEMENT

PROGRAM CONTACT:
John Phillips
301 5551212
john.phillips@nih.gov

(Privileged Communication)

Release Date: **02/19/2020**
Revised Date:

Application Number: **1 R01 AG068190-01**

Principal Investigator

SCHNEIDER, STEFAN

Applicant Organization: UNIVERSITY OF SOUTHERN CALIFORNIA

Review Group: **SSPA**
Social Sciences and Population Studies A Study Section

Meeting Date: **02/06/2020**
Council: **MAY 2020**
Requested Start: **07/01/2020**

RFA/PA: **PAR19-070**
PCC: **2CECNJP**

Project Title: **Testing early markers of cognitive decline and dementia derived from survey response behaviors**
SRG Action: **Impact Score:20**
Next Steps: **Visit https://grants.nih.gov/grants/next_steps.htm**
Human Subjects: **30-Human subjects involved - Certified, no SRG concerns**
Animal Subjects: **10-No live vertebrate animals involved for competing appl.**
Gender: **1A-Both genders, scientifically acceptable**
Minority: **1A-Minorities and non-minorities, scientifically acceptable**
Age: **3A-No children included, scientifically acceptable**

Project Year	Direct Costs Requested	Estimated Total Cost
1	499,976	817,894
2	498,535	815,537
3	498,759	815,903
4	499,597	817,274
5	499,662	817,380
TOTAL	2,496,529	4,083,989

ADMINISTRATIVE BUDGET NOTE: The budget shown is the requested budget and has not been adjusted to reflect any recommendations made by reviewers. If an award is planned, the costs will be calculated by Institute grants management staff based on the recommendations outlined below in the COMMITTEE BUDGET RECOMMENDATIONS section.

1R01AG068190-01 Schneider, Stefan

RESUME AND SUMMARY OF DISCUSSION: In this application, the investigators propose to use data from 16 longitudinal surveys from around the world to develop and validate new strategies for identifying preclinical markers of cognitive decline and dementia, by examining patterns of responses based on *how* people complete questionnaires rather than relying on the answers to specific questions. During discussion, the reviewers agreed the proposed project addresses a very timely topic with the aging of the Baby Boom population and will move the field forward in a significant way by developing a tool to measure early indicators of dementia that is more nimble and accessible than current tools. A critical strength is the innovative use of response style and paradata to extract measures of cognitive function and trajectory indirectly from characteristics of survey completion, even from surveys that do not have strong direct measures of cognitive function. The scientific premise is well-established and supported by compelling preliminary evidence. The Principal Investigator is a thought leader in this space and the rest of the team is exceptionally well trained. Other notable strengths include clever analytic strategies. The identified limitations are negligible; there are no major weaknesses. Overall, committee members agreed the proposed project would have a high impact on the field of cognitive aging research.

DESCRIPTION (provided by applicant): Discovering preclinical markers of cognitive and functional decline in mild cognitive impairment and dementia is fundamental for treatment development and to delay disease onset and progression. Subtle functional deficits on cognitively demanding activities often foreshadow dementia onset, but these early deficits are difficult to assess objectively with conventional methods. The proposed studies aspire to develop and validate performance-based indices for measuring functional deficits at older ages that are cost-effective, unobtrusive, and that could serve as early markers of subsequent cognitive decline and dementia. Specifically, we propose to develop indices of functional deficits that can be derived from participant response behaviors in existing population representative surveys. Completing a survey is a complex and cognitively demanding task that taxes a respondent's neuropsychological capacity. By focusing on how individuals complete surveys, we aim to derive a series of indices of functional deficits using two approaches: (1) The first approach consists of indices that are directly computed from participants' response patterns in questionnaires to capture invalid, incoherent, or erroneous responding on rating scales (examples include agreeing or disagreeing with statements regardless of content, skipping questions, or giving contradictory responses). (2) The second approach considers indices derived from individuals' computer use behavior in online surveys to measure the efficiency, speed, and consistency of behaviors during the completion of online surveys (examples include the proportion of corrected/changed answers, average response time, and response time variability). To evaluate the validity and clinical utility of the indices, we will systematically examine their associations with conceptually related constructs (concurrent cognitive test scores, instrumental activities of daily living, financial wellbeing, frailty), their sensitivity to change with age, their ability to predict subsequent cognitive decline, and their ability to predict the subsequent onset of mild cognitive impairment and dementia. Self-report surveys administered regularly in 16 existing longitudinal panel studies (>50,000 participants) will provide a rich basis for developing and testing indices derived from response patterns in questionnaires. An ongoing population representative Internet panel will provide the opportunity to test computer use behavior indices that are unobtrusively recorded “in the background” of online surveys. Marshalling multiple datasets and aggregating results across diverse samples and survey measures using identical data-analytic models will greatly enhance generalizability and test the breadth of applicability of each index. Examining the predictive accuracy of the indices alone and in concert will allow us to identify those indices that contribute substantial prognostic information and those that provide irrelevant or redundant information. This research has potential to broaden the repertoire of

available tools that could signal cognitive and functional decline in older ages and allow for advanced study of dementia.

PUBLIC HEALTH RELEVANCE: Dementia is a significant public health concern. The proposed studies aspire to develop and validate new strategies for identifying preclinical markers of cognitive decline and dementia based on the ways in which people complete questionnaires in population representative surveys. This research has the potential to enable early detection of dementia with tools that are cost-effective, unobtrusive, and scalable for use in large samples, allowing advanced study of the disease.

CRITIQUE 1

Significance: 2
Investigator(s): 2
Innovation: 1
Approach: 2
Environment: 1

Overall Impact: Dementia is a significant public health concern. The proposed studies aspire to develop and validate new strategies for identifying preclinical markers of cognitive decline and dementia based on the ways in which people complete questionnaires in population representative surveys. The proposed project is characterized by its strengths across the board. Minor to moderate weaknesses in significance, investigators, and approach dampen enthusiasm minimally. In general, the study's strengths outweigh its weaknesses, suggesting that it has a high likelihood of exerting a sustained and powerful influence on our understanding of cognitive and functional decline in older ages, thus allowing for advanced study of dementia.

1. Significance:

Strengths

- Any focus on the causes, consequences, treatment, and impacts of dementia is important given the disease's high prevalence, the health care costs associated with its treatment, and the burden it places on individuals, families, and societies.
- The aging baby boomer generation, coupled with increases in life expectancy, underscores the urgency of tangible progress in understanding this disease.
- The project seeks to develop methods of detecting preclinical dementia using cost-effective and unobtrusive survey-based indices. The rigorously-executed prior research (including pilot work undertaken by members of the proposed team) supports the notion that alterations in daily functioning, such as how people complete surveys, predicts cognitive decline and dementia.
- Existing measures designed to detect preclinical dementia are often impracticable as they are either biologically-based or taxing on respondents and/or costly due to the specialized time and equipment involved. Development of a cost-effective, unobtrusive, and scalable methods would address a critical barrier in the field.
- The project is highly responsive to calls from the NIA and the Alzheimer's Association to identify and validate new neurobehavioral measures to detect preclinical manifestations of dementia and mild cognitive impairment.

Weaknesses

- It is unclear how the results of the study will or can be used to improve clinical practice. Given that the applicant states that the utility of the study is that early, tailored intervention can be facilitated, more needs to be said about how the survey-based indices can be used to intervene rather than just detect or describe.

2. Investigator(s):

Strengths

- PI Schneider is a thought leader in the development of methods to identify bias in self-reports and techniques to augment what can be learned about individuals from their self-report behaviors. His publication record is commendable and his track record of securing extramural support for his work as MPI, PI, or Co-I positions him well for the proposed undertaking.
- The balance of the study team is exceptionally well-trained, productive, and impactful in the survey methods field. Much of what is known about how people respond to surveys and what can be learned by looking at how they answer questions (rather than the content of their answers) has been contributed by members of the team.
- The bench of collaborators skilled in psychometric analyses and expert in the area of dementia is deep in this group.
- Select members of the team, particularly PI Schneider and Co-Is Stone and Junghaenel , have collaborated together successfully in the past.

Weaknesses

- The number of Co-Is is quite high and a few possess a fair amount of overlapping expertise.
- Even though Co-I Langa is trained as a physician and possesses expertise in cognitive function, mild cognitive impairment, and dementia, it is unclear whether he still practices medicine. Addition of a practicing geriatrician with direct experience treating patients with dementia would provide a different lens to the work, better guide the conduct of the study, facilitate the interpretation of results, and smooth the path to clinical application.

3. Innovation:

Strengths

- The use of survey-response style/behavior in traditional questionnaires and paradata in online surveys to detect preclinical dementia is the study's primary innovation.
- Leveraging multiple data sets and aggregating results across diverse samples and survey measures using identical data analytic models is novel and will undergird the inferential value and portability of the results.
- Many of the data collection and statistical methods are rather standard in the field but they are creatively deployed in the proposed study.

Weaknesses

- None noted.

4. Approach:

Strengths

- The preliminary studies undertaken by members of the study team, when coupled with the extant literature in this area, offer proof-of-principle evidence that strengthens the positive assessment of the approach's scientific rigor.
- Use of a very large number (n=16) of longitudinal studies will decrease the likelihood that results are idiosyncratic to a particular survey or population. The representation of a range of different cognitive assessments (clinical and non-clinical) in each survey will similarly augment the robustness of the findings.
- Building on the infrastructure of the Understanding America Study (UAS) allows for longitudinal collection and assessment of computer-based paradata (e.g., response latencies).
- The use of both response styles in traditional surveys and online survey paradata allows for a broader set of analyses and inferences.
- The response style- and paradata-derived indices are well-described and justified and supported by a body of published work in this area.
- Construction of a "Frailty Index" will allow for a more parsimonious analyses and interpretation, while limiting the need for multiple comparison corrections like Bonferroni.
- Analyzing each data set separately using identical data analytic models then synthesizing results using meta-analytic methods is clever.
- The use of feature selection/machine learning allows the team to ascertain an optimal set of indices that can be used to predict dementia.
- The applicant allotted 1.5 years to the process of data acquisition and preparation. This is realistic and reflects an understanding of the practical aspects of work of this nature and scope. It also speaks to the feasibility of the study and justifies the five year grant period.
- Overall, the analytic strategy is sound and the scientific rigor is high.

Weaknesses

- By design, the project focuses on survey respondents. Some discussion of whom might be excluded from the analyses because of nonresponse in terms of age and cognitive status (at the very least) should be offered along with a discussion of what their exclusion might mean for the generalizability of the findings.
- Some initial letters of support from the principals of the 16 surveys to be used, with a specific nod towards a willingness to support data use agreements, would have increased confidence that these data sources can actually be used.

5. Environment:

Strengths

- The research infrastructure at USC are excellent and should support the needs of the study.

Weaknesses

- None noted.

Protections for Human Subjects:

Acceptable Risks and/or Adequate Protections

Data and Safety Monitoring Plan (Applicable for Clinical Trials Only):

Not Applicable (No Clinical Trials)

Inclusion Plans:
- Sex/Gender: Distribution justified scientifically
- Race/Ethnicity: Distribution justified scientifically
- For NIH-Defined Phase III trials, Plans for valid design and analysis:
- Inclusion/Exclusion Based on Age: Distribution justified scientifically

Vertebrate Animals:
Not Applicable (No Vertebrate Animals)

Biohazards:
Not Applicable (No Biohazards)

Applications from Foreign Organizations:
Not Applicable (No Foreign Organizations)

Select Agents:
Not Applicable (No Select Agents)

Resource Sharing Plans:
Acceptable

Authentication of Key Biological and/or Chemical Resources:
Not Applicable (No Relevant Resources)

Budget and Period of Support:
Recommend as Requested

CRITIQUE 2

Significance: 1
Investigator(s): 1
Innovation: 2
Approach: 3
Environment: 1

Overall Impact: This proposal would like to study whether *how* one responds to surveys, as opposed to the *answers* to survey questions, can predict MCI and dementia. They will bring numerous (16) preexisting longitudinal data sets to bear on this question, domestic and international, with a broad set

of questions and topics covered. Aim 2 will study how people use computers as a potential predictor for MCI and dementia. Cognitive function will be measured in a variety of ways, depending on the survey instrument. Dementia will be measured by diagnosis, and pre-specified cognition cut-offs, depending on survey instrument. Due to the differences between surveys, they propose Individual Participant Data meta-analysis, and not data harmonization. They will use a variety of regression techniques (logistic regressions, survival analysis) to test for the temporal correlation and longitudinal changes in cognition. Then they will select the optional combination of features that are most predictive.

This proposal is scientifically rigorous and innovative in its proposed indices for measuring cognitive function and changes in cognitive function. The greatest weakness of the proposal is the variation in cognitive function measures in the existing datasets and the degree to which these measures are sensitive/specific, especially for minorities or low-education groups. This is largely a weakness of the current science, not specific to this proposal, but the investigators could be more sensitive to this issue in terms of training algorithms that may have biases already built in. Overall, this proposal has the potential to have a significant impact on the field.

1. Significance:

Strengths

- Current screening mechanisms for dementia have either low specificity/sensitivity or are timely/costly to administer. The proposal has, instead, cost-effective, unobtrusive, and scalable tests.
- Relatively easily implementable if proven helpful.
- Identifying individuals predicted to have cognitive decline could help with research as well as identifying intervention points, once we have effective interventions.

Weaknesses

- Unclear whether these are perfectly transferable measures to surveys taken in other forums (such as the doctor's office) as investigator's point out.

2. Investigator(s):

Strengths

- Team is strong, and has worked together

Weaknesses

- None noted

3. Innovation:

Strengths

- Current screening mechanisms for dementia have either low specificity/sensitivity or are timely/costly to administer. The proposal has, instead, cost-effective, unobtrusive, and scalable tests.
- Methods are appropriate for the data at hand.

- Has the potential to provide predicted cognitive measures across other surveys that have the indices for *how* the survey was answered, even without cognition measures.

Weaknesses

- The methods themselves used to answer the questions at hand are appropriate, but not necessarily innovative.

4. Approach:

Strengths

- Preliminary studies with the HRS are promising.
- Preliminary studies with the UAS panel, age 50+ is also promising, especially with the levels of cognitive function (less so with the change, but still there).

Weaknesses

- Cognitive function is hard to measure and survey measures are themselves a proxy for cognition.
- Different measures of cognitive function are available across surveys
- Dementia diagnosis only available in 10 studies.
- Concerns with differences in cognitive function tests for minorities and low-education individuals are rampant.

5. Environment:

Strengths

- USC is a great place for this type of research.

Weaknesses

- None noted

Protections for Human Subjects:

Acceptable Risks and/or Adequate Protections

Inclusion Plans:

- Sex/Gender: Distribution justified scientifically
- Race/Ethnicity: Distribution justified scientifically
- For NIH-Defined Phase III trials, Plans for valid design and analysis:
- Inclusion/Exclusion Based on Age: Distribution justified scientifically

Vertebrate Animals:

Not Applicable (No Vertebrate Animals)

Biohazards:

Not Applicable (No Biohazards)

Applications from Foreign Organizations:
Not Applicable (No Foreign Organizations)

Select Agents:
Not Applicable (No Select Agents)

Resource Sharing Plans:
Acceptable

Authentication of Key Biological and/or Chemical Resources:
Not Applicable (No Relevant Resources)

Budget and Period of Support:
Recommend as Requested

CRITIQUE 3

Significance: 1
Investigator(s): 3
Innovation: 1
Approach: 2
Environment: 3

Overall Impact: This R01 from an experienced researcher with relevant prior grants and papers addresses a high priority research goal: extracting additional information about cognitive trajectories and risk from settings where cognition was not directly assessed. They pursue this using (aim 1) data from several major survey studies of aging and (aim 2) data from an online survey panel, all of which include concurrently measured cognitive assessments to allow training and validation of prediction models based on information like skip patterns, don't know answers, random errors, response delays. This is an under-researched area, so the proposal is innovative; the methods are strong, using appropriate machine learning and validation methods for a prediction model. The only minor weaknesses are lack of clarity that they can access all of the survey data necessary for Aim 1; concerns about feasibility given the distribution of the budget towards high-level researchers; and ambiguity about disentangling physical and cognitive outcomes. The overall impact of this research is high because the findings will be used by many other researchers as well as potentially in clinical settings (with modification).

1. Significance:

Strengths

- Extracting measures of cognitive function and trajectory indirectly from characteristics of survey completion or computer could have tremendous importance for research (allowing us to

understand how cognition is changing across the lifecourse, from surveys that never collected good cognitive measures) and for clinical care (identifying people who may be experiencing subtle cognitive changes).

- Computational advances have made this potential much more feasible in recent years.
- Whether positive or null, these findings will be valuable.

Weaknesses

- None noted by the reviewer.

2. Investigator(s):

Strengths

- PI is a social psychologist with extensive experience in measurement including work extracting information from survey meta- data or indirect features of survey responses.
- Junghaenel is a social psychologist and will be project director
- Consultant Langa has expertise in and surveys from his long-time leadership in HRS.
- Stone is a clinical psychologist with expertise on patient reported outcomes and ecological momentary assessment
- Zelinski provides expertise in cognition and is PI of one of the studies to be used (the very small Longbeach Longitudinal Study).
- Meijer is a psychometrician and econometrician
- Angrisani is an econometrician w/ expertise in survey methodology/weighting
- Orriens is the IT director and will extract the paradata from the online survey
- Jin is a systems engineer and will oversee the statistical learning models
- Kapteyn is director of the online survey panel

Weaknesses

- Budget has a lot of senior people who will provide perhaps opinions and wisdom but probably not do much hands-on analytic work, whereas this is a very intensive analysis project. Some people are on for a minimal amount of effort (Kapteyn) and will clearly contribute but others have fairly high efforts for the amount of time they are likely to be able (or needed) to commit. This is a particular issue because it seems to have pushed out budget for more scientific data analysis staff or more early career researchers likely to be in the weeds with the data.

3. Innovation:

Strengths

- Use of indirect information extracted from questionnaires is certainly happening in industry and a very little exploration in academic research, but this is the most systematic and ambitious effort I have seen.

Weaknesses

- None noted by the reviewer.

4. Approach:

Strengths

- Multiple surveys allowing for comparison of patterns
- Longitudinal and cross-sectional evaluations
- Online and CaPI format; attention to mode of assessment
- Use of elastic nets and appropriate statistical approaches (e.g. cross-validation) for identifying an optimal prediction model

Weaknesses

- All weaknesses were minor and some relate to work I would like them to have included but could arguably be considered too ambitious for this single grant.
- Unclear how they will address incomplete or missing data (when that is not the indicator itself)
- Uncertain they will be able to access all needed data from all the surveys. Langa's participation suggests they will be able to access meta-data and anything necessary from HRS; same for Long Beach due to Zelinski's participation, but others are uncertain.
- This is the type of setting where quirks of the study process in the field might be very important. It would therefore be valuable to have other people directly involved in each of the surveys involved in this project to some extent (e.g., as consultants or as an advisory board). This might be not just the PIs of those studies but directors of field operations who might be more familiar with year to year variations in process.
- The frailty index is non-specific and conceptually fuzzy. It is not realistic to consider that it operates equally across all surveys.
- There will be an important challenge in disentangling cognition from physical deterioration for some measures, so discriminant validity would be important.
- The data are proposed to be transformed to have weak stationarity, consistent with typical time series analyses, but in this case weak stationarity seems inconsistent with the very conception of aging, and I fear this transformation may obscure important age related changes in average functioning and heterogeneity of functioning.

5. Environment:

Strengths

- USC is a hub for this type of research and will be a strong setting.

Weaknesses

- None noted by the reviewer.

Protections for Human Subjects:

Not Applicable (No Human Subjects)

Data and Safety Monitoring Plan (Applicable for Clinical Trials Only):

Not Applicable (No Clinical Trials)

Inclusion Plans:

- Sex/Gender: Distribution justified scientifically
- Race/Ethnicity: Distribution justified scientifically
- For NIH-Defined Phase III trials, Plans for valid design and analysis:
- Inclusion/Exclusion Based on Age:
- Using everyone available in multiple representative studies

Vertebrate Animals:
Not Applicable (No Vertebrate Animals)

Biohazards:
Not Applicable (No Biohazards)

Applications from Foreign Organizations:
Not Applicable (No Foreign Organizations)

Select Agents:
Not Applicable (No Select Agents)

Resource Sharing Plans:
Acceptable

- Strong data sharing protocol. Since the main resource here will be code for creating the measures, it would be important to post that as well.

Authentication of Key Biological and/or Chemical Resources:
Not Applicable (No Relevant Resources)

Budget and Period of Support:
Recommend as Requested

THE FOLLOWING SECTIONS WERE PREPARED BY THE SCIENTIFIC REVIEW OFFICER TO SUMMARIZE THE OUTCOME OF DISCUSSIONS OF THE REVIEW COMMITTEE, OR REVIEWERS' WRITTEN CRITIQUES, ON THE FOLLOWING ISSUES:

PROTECTION OF HUMAN SUBJECTS: ACCEPTABLE

INCLUSION OF WOMEN PLAN: ACCEPTABLE

INCLUSION OF MINORITIES PLAN: ACCEPTABLE

INCLUSION ACROSS THE LIFESPAN PLAN: ACCEPTABLE

COMMITTEE BUDGET RECOMMENDATIONS: The budget was recommended as requested.

Footnotes for 1 R01 AG068190-01; PI Name: Schneider, Stefan

NIH has modified its policy regarding the receipt of resubmissions (amended applications). See Guide Notice NOT-OD-14-074 at http://grants.nih.gov/grants/guide/notice-files/NOT-OD-14-074.html. The impact/priority score is calculated after discussion of an application by averaging the overall scores (1-9) given by all voting reviewers on the committee and multiplying by 10. The criterion scores are submitted prior to the meeting by the individual reviewers assigned to an application, and are not discussed specifically at the review meeting or calculated into the overall impact score. Some applications also receive a percentile ranking. For details on the review process, see http://grants.nih.gov/grants/peer_review_process.htm#scoring.

MEETING ROSTER

Social Sciences and Population Studies A Study Section
Population Sciences and Epidemiology Integrated Review Group
CENTER FOR SCIENTIFIC REVIEW
SSPA
02/06/2020 - 02/07/2020

Notice of NIH Policy to All Applicants: Meeting rosters are provided for information purposes only. Applicant investigators and institutional officials must not communicate directly with study section members about an application before or after the review. Failure to observe this policy will create a serious breach of integrity in the peer review process, and may lead to actions outlined in NOT-OD-14-073 at https://grants.nih.gov/grants/guide/notice-files/NOT-OD-14-073.html and NOT-OD-15-106 at https://grants.nih.gov/grants/guide/notice-files/NOT-OD-15-106.html, including removal of the application from immediate review.

CHAIRPERSON(S)

HARRIS, KATHLEEN MULLAN, PHD
JAMES HAAR DISTINGUISHED PROFESSOR
DEPARTMENT OF SOCIOLOGY
UNIVERSITY OF NORTH CAROLINA AT CHAPEL HILL
CHAPEL HILL, NC 27516

MEMBERS

BEEBE, TIMOTHY, PHD
MAYO PROFESSOR AND DIVISION HEAD
DIVISON OF HEALTH POLICY AND MANAGEMENT
SCHOOL OF PUBLIC HEALTH
UNIVERSITY OF MINNESOTA
MINNEAPOLIS, MN 55455

BENJAMIN, DANIEL J, PHD *
PROFESSOR (RESEARCH) OF ECONOMICS
CENTER FOR ECONOMIC AND SOCIAL RESEARCH
UNIVERSITY OF SOUTHERN CALIFORNIA
LOS ANGELES, CA 90089

BRUCKNER, TIM ALLEN, PHD *
ASSOCIATE PROFESSOR
DEPARTMENT OF PUBLIC HEALTH
UNIVERSITY OF CALIFORNIA, IRVINE
IRVINE, CA 92697

CAGNEY, KATHLEEN A, PHD
PROFESSOR OF SOCIOLOGY
DEPUTY DEAN
DIVISION OF SOCIAL SCIENCES
UNIVERSITY OF CHICAGO
CHICAGO, IL 60637

CLARKE, PHILIPPA, PHD *
PROFESSOR
DEPARTMENT OF EPIDEMIOLOGY
UNIVERSITY OF MICHIGAN
ANN ARBOR, MI 48104

COE, NORMA B, PHD *
ASSOCIATE PROFESSOR
DEPARTMENT OF MEDICAL ETHICS AND HEALTH POLICY
PERELMAN SCHOOL OF MEDICINE
UNIVERSITY OF PENNSYLVANIA
PHILADELPHIA, PA 19105

DAVE, DHAVAL M, PHD
STANTON PROFESSOR OF ECONOMICS
NATIONAL BUREAU OF ECONOMIC RESEARCH
BENTLEY UNIVERSITY
WALTHAM, MA 02452

GIOVENCO, DANIEL PHILIP, PHD *
ASSISTANT PROFESSOR
DEPARTMENT OF SOCIOMEDICAL SCIENCES
MAILMAN SCHOOL OF PUBLIC HEALTH
COLUMBIA UNIVERSITY
NEW YORK, NY 10032

GLYMOUR, MEDELLENA MARIA, SCD
PROFESSOR
DEPARTMENT OF EPIDEMIOLOGY AND BIOSTATISTICS
UNIVERSITY OF CALIFORNIA, SAN FRANCISCO
SAN FRANCISCO, CA 94158

GRUCZA, RICHARD A, PHD
PROFESSOR
DEPARTMENT OF FAMILY AND COMMUNITY MEDICINE
CENTER FOR HEALTH OUTCOMES RESEARCH
SAINT LOUIS UNIVERSITY
SAINT LOUIS, MO 63103

HALPERN-FELSHER, BONNIE L, PHD
PROFESSOR
DEPARTMENT OF PEDIATRICS
DIVISION OF ADOLESCENT MEDICINE
STANFORD UNIVERSITY SCHOOL OF MEDICINE
PALO ALTO, CA 94304

HANDA, SUDHANSHU, PHD
LAWRENCE I GILBERT DISTINGUISHED PROFESSOR
DEPARTMENT OF PUBLIC POLICY
UNIVERSITY OF NORTH CAROLINA AT CHAPEL HILL
CHAPEL HILL, NC 27599

HERD, PAMELA, PHD
PROFESSOR
MCCOURT SCHOOL OF PUBLIC POLICY
GEORGETOWN UNIVERSITY
WASHINGTON, DC 20057

HOWARD, TIMOTHY D, PHD *
PROFESSOR
CENTER FOR GENOMICS AND
PERSONALIZED MEDICINE RESEARCH
WAKE FOREST SCHOOL OF MEDICINE
WINSTON-SALEM, NC 27157

LINK, BRUCE G, PHD *
DISTINGUISHED PROFESSOR OF PUBLIC POLICY AND
SOCIOLOGY
SCHOOL OF PUBLIC POLICY
UNIVERSITY OF CALIFORNIA, RIVERSIDE
RIVERSIDE 92521

LIU, HUI, PHD *
PROFESSOR
DEPARTMENT OF SOCIOLOGY
MICHIGAN STATE UNIVERSITY
EAST LANSING, MI 48824

LOGAN, JOHN R, PHD
PROFESSOR
DEPARTMENT OF SOCIOLOGY
BROWN UNIVERSITY
PROVIDENCE, RI 02912

MAGNUSON, KATHERINE, PHD *
PROFESSOR
SCHOOL OF SOCIAL WORK
UNIVERSITY OF WISCONSIN-MADISON
MADISON, WI 53706

MAGZAMEN, SHERYL, PHD *
ASSOCIATE PROFESSOR
DEPARTMENT OF ENVIRONMENTAL
AND RADIOLOGICAL HEALTH SCIENCES
COLORADO STATE UNIVERSITY
FORT COLLINS, CO 80523

MARSHALL, GILLIAN L, PHD *
ASSISTANT PROFESSOR
DEPARTMENT OF SOCIAL WORK
UNIVERSITY OF WASHINGTON
SEATTLE, WA 98105

NEELON, BRIAN, PHD *
ASSOCIATE PROFESSOR
DEPARTMENT OF PUBLIC HEALTH SCIENCES
MEDICAL UNIVERSITY OF SOUTH CAROLINA
CHARLESTON, SC 29425

PERRY, BREA LOUISE, PHD
PROFESSOR
DEPARTMENT OF SOCIOLOGY
INDIANA UNIVERSITY
BLOOMINGTON, IN 47405

RICHARDSON, GALE A., PHD *
PROFESSOR
DEPARTMENT OF PSYCHIATRY AND EPIDEMIOLOGY
UNIVERSITY OF PITTSBURGH
PITTSBURGH, PA 15213

SHIH, REGINA A, PHD
SENIOR BEHAVIORAL AND SOCIAL SCIENTIST
RAND CORPORATION
ARLINGTON, VA 22202

SIBILLE, KIMBERLY THERESA, PHD *
ASSOCIATE PROFESSOR
DEPARTMENT OF AGING AND
GERIATRIC RESEARCH
COLLEGE OF MEDICINE
UNIVERSITY OF FLORIDA
GAINESVILLE 32611

SLOPEN, NATALIE B, DSC *
ASSISTANT PROFESSOR
DEPARTMENT OF EPIDEMOLOGY AND BIOSTATISTICS
UNIVERSITY OF MARYLAND, COLLEGE PARK
COLLEGE PARK, MD 201742

VUOLO, MICHAEL C, PHD *
ASSOCIATE PROFESSOR
DEPARTMENT OF SOCIOLOGY
THE OHIO STATE UNIVERSITY
COLUMBUS, OH 43210

YANG, YONG, PHD *
ASSISTANT PROFESSOR
DIVISION OF SOCIAL AND BEHAVIORAL SCIENCES
SCHOOL OF PUBLIC HEALTH
UNIVERSITY OF MEMPHIS
MEMPHIS, TN 38117

YOUNT, KATHRYN M, PHD *
ASA GRIGGS CANDLER CHAIR AND PROFESSOR
HUBERT DEPARTMENT OF GLOBAL HEALTH
ROLLINS SCHOOL OF PUBLIC HEALTH
EMORY UNIVERSITY
ATLANTA, GA 30322

SCIENTIFIC REVIEW OFFICER

RYAN, SUZANNE, PHD
SCIENTIFIC REVIEW OFFICER
CENTER FOR SCIENTIFIC REVIEW
NATIONAL INSTITUTES OF HEALTH
BETHESDA, MD 20892

ZENA, SAMRAWIT
EXTRAMURAL SUPPORT ASSISTANT
INFECTIOUS DISEASES AND MICROBIOLOGY IRG
CENTERS FOR SCIENTIFIC REVIEW
NATIONAL INSTITUTES OF HEALTH
BETHESDA, MD 20892

* Temporary Member. For grant applications, temporary members may participate in the entire meeting or may review only selected applications as needed.

Consultants are required to absent themselves from the room during the review of any application if their presence would constitute or appear to constitute a conflict of interest.
